# SARS-CoV-2 seroprevalence survey among health care providers in a Belgian public multiple-site hospital

**DOI:** 10.1017/S0950268821001497

**Published:** 2021-08-10

**Authors:** Reinout Naesens, Helena Mertes, Johan Clukers, Sereina Herzog, Christiane Brands, Philippe Vets, Inneke De laet, Peggy Bruynseels, Pieter De Schouwer, Sanne van der Maas, Katrien Bervoets, Niel Hens, Pierre Van Damme

**Affiliations:** 1 Department of Medical Microbiology, ZiekenhuisNetwerk Antwerpen, B-2020Antwerpen, Belgium; 2 Department of Infection Prevention and Control, ZiekenhuisNetwerk Antwerpen, B-2020Antwerpen, Belgium; 3 Department of Infectious Disease, ZiekenhuisNetwerk Antwerpen, B-2020Antwerpen, Belgium; 4 Department of Respiratory Medicine, ZiekenhuisNetwerk Antwerpen, B-2020Antwerpen, Belgium; 5 Centre for Health Economics Research and Modelling of Infectious Diseases (CHERMID), Vaccine & Infectious Disease Institute (VAXINFECTIO), University of Antwerp, B-2610Wilrijk, Belgium; 6 Department of Intensive Care and Anesthesiology, ZiekenhuisNetwerk Antwerpen, Antwerpen, Belgium; 7 Hospital and Medical Directory Board, ZiekenhuisNetwerk Antwerpen, B-2020Antwerpen, Belgium; 8 Data Science Institute, I-BioStat, UHasselt, B-3500Hasselt, Belgium; 9 Centre for the Evaluation of Vaccination, Vaccine and Infectious Disease Institute, University of Antwerp, B-2610Wilrijk, Belgium

**Keywords:** SARS-CoV-2, seroprevalence, health care worker, risk factor, professional category

## Abstract

Although the severe acute respiratory syndrome coronavirus 2 (SARS-CoV-2) pandemic is lasting for more than 1 year, the exposition risks of health-care providers are still unclear. Available evidence is conflicting. We investigated the prevalence of antibodies against SARS-CoV-2 in the staff of a large public hospital with multiple sites in the Antwerp region of Belgium. Risk factors for infection were identified by means of a questionnaire and human resource data. We performed hospital-wide serology tests in the weeks following the first epidemic wave (16 March to the end of May 2020) and combined the results with the answers from an individual questionnaire. Overall seroprevalence was 7.6%. We found higher seroprevalences in nurses [10.0%; 95% confidence interval (CI) 8.9–11.2] than in physicians 6.4% (95% CI 4.6–8.7), paramedical 6.0% (95% CI 4.3–8.0) and administrative staff (2.9%; 95% CI 1.8–4.5). Staff who indicated contact with a confirmed coronavirus disease 2019 (COVID-19) colleague had a higher seroprevalence (12.0%; 95% CI 10.7–13.4) than staff who did not (4.2%; 95% CI 3.5–5.0). The same findings were present for contacts in the private setting. Working in general COVID-19 wards, but not in emergency departments or intensive care units, was also a significant risk factor. Since our analysis points in the direction of active SARS-CoV-2 transmission within hospitals, we argue for implementing a stringent hospital-wide testing and contact-tracing policy with special attention to the health care workers employed in general COVID-19 departments. Additional studies are needed to establish the transmission dynamics.

## Introduction

Since the beginning of the severe acute respiratory syndrome coronavirus 2 (SARS-CoV-2) coronavirus disease 2019 (COVID-19) pandemic in December 2019, the infection has been responsible for more than 170 million cases and more than 3 million deaths worldwide as of May 2021 [1].

Health care providers (HCPs) are on the frontline where there is remaining uncertainty about adequate management of personal protective equipment (PPE) [2–4]. Literature regarding the increased risks in HCPs is conflicting. Several serosurveys have been published showing that seropositivity in HCPs is not related to healthcare occupation, workplace factors, or contact with patients with known COVID-19 [5–8]. In those surveys, significant associations were only found with community exposure or exposure in the private setting. Other studies and epidemiological reports showed increased risks in HCPs, even where there is adequate availability of PPE [9–16].

**Fig. 1.**
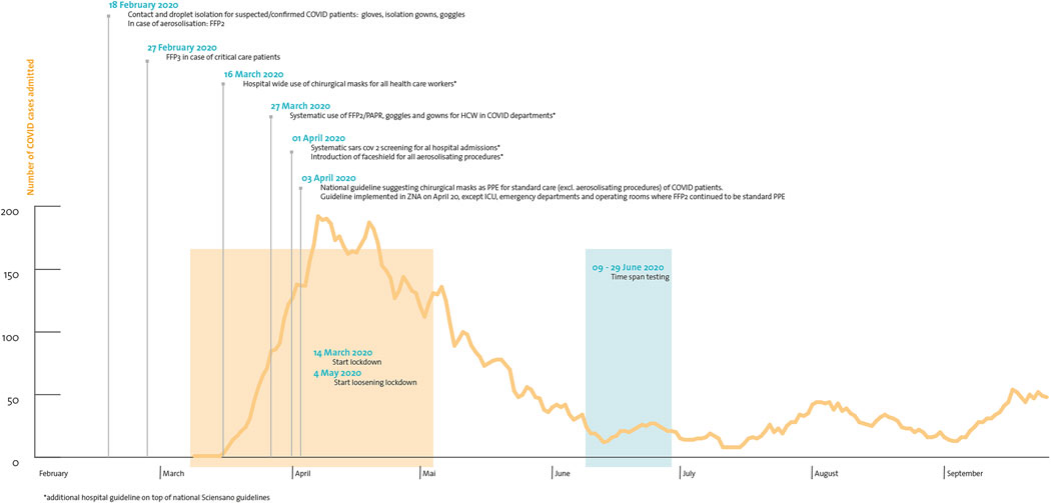
Number of admitted patients in ZNA in relation with population and hospital precautions.

The hospital network of Antwerp (Ziekenhuisnetwerk Antwerpen, ZNA) is a public multiple-site hospital, currently the largest in Belgium. It comprises three acute care hospitals [each with an emergency department (ED) and intensive care unit (ICU)], a children's hospital and five more chronic care facilities, with a total capacity of 2500 hospital beds. During the first COVID-19 epidemic wave, ZNA was highly impacted and accounted for substantial numbers of COVID-19 admissions (Fig. 1).

At the beginning of the COVID-19 pandemic, a Scientific Board was established at ZNA, which comprised microbiologists, infection prevention and infectious disease specialists, pneumologists and intensive care specialists, to advise the Hospital Direction Board. For infection prevention precautions, Belgian guidelines (Sciensano) were followed strictly [17]. Additionally, hospital-wide use of surgical masks was introduced on 16 March for all HCPs, and systematic testing of all patients on hospital admission was introduced on 1 April by SAR-CoV-2 reverse transcription-polymerase chain reaction (RT-PCR). This was earlier than the testing policy issued by the Belgian governmental scientific service (Sciensano) [17] (Fig. 1).

We conducted a voluntary hospital-wide SARS-CoV-2 serology survey from 9 June to 29 June 2020 and combined the results with the answers from a questionnaire that was sent to all hospital staff in the weeks following the first epidemic wave in Belgium (16 March to the end of May 2020).

The aim of the study was to determine seroprevalence of SARS-CoV-2 in HCPs and identify potential risk factors (type of ward, job category, (un)protected contact with positive cases and contact with positive cases in the private setting) associated with (a)symptomatic infection with SARS-CoV-2, in order to optimise hospital strategies and procedures.

## Methods

From 9 June to 29 June 2020, all 6838 employees working at ZNA were offered a serological test. HCPs included all hospital staff (nurses, doctors, paramedics, administrative and maintenance staff). The serology test was only performed if the questionnaire (with questions concerning possible risk factors for exposure to SARS-CoV-2) was completed. Questions covered the period from 1 March to the moment of the serology test.

The questionnaire asked for time spent (<1week, 1–2 weeks, 3–4 weeks and >4 weeks) in different COVID-19 departments. COVID-19 departments consisted of suspected COVID-19 units (suspected COVID-19 patients on general wards), confirmed COVID-19 units (confirmed COVID-19 cases on general wards), COVID-19 ED (suspected/confirmed cases presenting in the ED) and COVID-19 ICUs (suspected and confirmed COVID-19 cases in the ICU).

The following risk factors were included in the questionnaire: contact with persons in the private or household setting with confirmed or suspected COVID-19 and/or the indication that a close colleague at work had confirmed COVID-19. We also asked for potential contact with positive COVID-19 patients (suspected or confirmed), thereby applying hospital guidelines on PPE (Fig. 1). Furthermore, accidental contacts with COVID-19 patients (thus not following hospital guidelines on PPE) (suspected or confirmed) was also registered in the questionnaire. No details on timing, frequency or duration of these contacts were registered.

We used an electrochemiluminescence immunoassay test from Roche for the qualitative detection of total IgM and IgG SARS-CoV-2 antibodies. The manufacturer reported a sensitivity of 95% and a specificity of 99%.

All data were collected and combined with a human resource database which delivered date of birth, domicile and professional categories. All combined data were encoded anonymously in an Access-database. The database was transferred by a data protection officer to the University of Antwerp which functioned as a trusted third party.

Seroprevalence estimates are displayed with exact Clopper−Pearson 95% confidence intervals (CIs). Data from all participating hospital staff were used for the analyses, with the following exception: analyses concerning the time spent on different COVID-19 departments were restricted to data of hospital staff effectively having patient contacts (e.g. nurses, doctors or paramedics, but not administrative staff).

Logistic linear mixed models with a random intercept for the hospital sites were used to investigate association between anti-SARS-CoV-2 IgG seropositivity and work related risk factors as well as with age and sex. The random intercept accounts for the potential centre effects by the different hospital sites. All associations were first assessed in univariate models. A stepwise model selection was performed, comparing the Akaike information criterion (AIC) of each model at a 5% significance level, including possible interaction, to determine the goodness of fit [18].

All analyses were performed using the statistical software R (version 4.0.3) {https://www.R-project.org/} using glmer() from lme4 package for the logistic linear mixed models and binom.test() for the seroprevalence estimates. The R code is available from the authors by request.

The data that support the findings of this study are available from ZNA upon request.

Each participant signed an informed consent form. Approval was obtained from the local Institutional Board Review (Approval N° 5383).

## Results

A total of 5233 individuals aged between 17 and 73 years (female 79.8%, male 20.2%) participated in the seroprevalence survey. This number represents (76.5%) of the hospital staff and the participation rate was >70% at all different hospital sites. The study population (i.e. participants) was representative to the overall hospital workforce regarding age, sex and professional categories. 79.8% of the study population were female, whereas 20.2% were male. Of the participants, 397 [7.6%; 95% confidence interval (CI) 6.9–8.3] tested positive for SARS-CoV-2 antibodies. Seroprevalences in male and female participants were respectively 7.0% (95% CI 5.5–8.7) and 7.7% (95% CI 6.9–8.6). Including only medical, nursing and paramedical staff with patient contacts, the seroprevalence was 8.7% (95% CI 7.9–9.7).

**Fig. 2.**
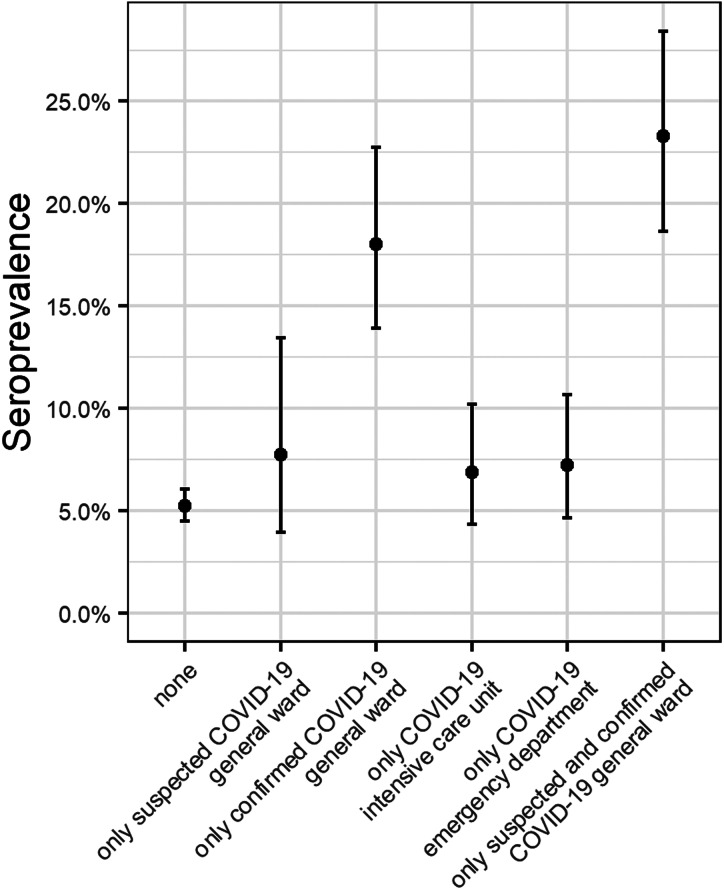
Seroprevalence in wards (not all combinations are shown).

Among the 5233 participants, 1944 (37.1%) worked on a COVID-19 ward including suspected COVID-19 wards, confirmed COVID-19 wards, COVID-19 ED and COVID-19 ICU. Seroprevalence in these HCPs was significantly higher (11.6%; 95% CI 10.2–13.1) than for individuals not generally working on a COVID-19 ward (5.2%; 95% CI 4.5–6). This observation is the same over all hospital sites (acute and chronic). Among those working on a COVID-19 ward, only HCPs working on a confirmed COVID-19 ward had higher seroprevalence (18.0%; 95% CI 13.9–22.7) than HCPs who only worked on suspected COVID-19 wards, COVID-19 ED or COVID-19 ICU (Fig. 2).

**Fig. 3.**
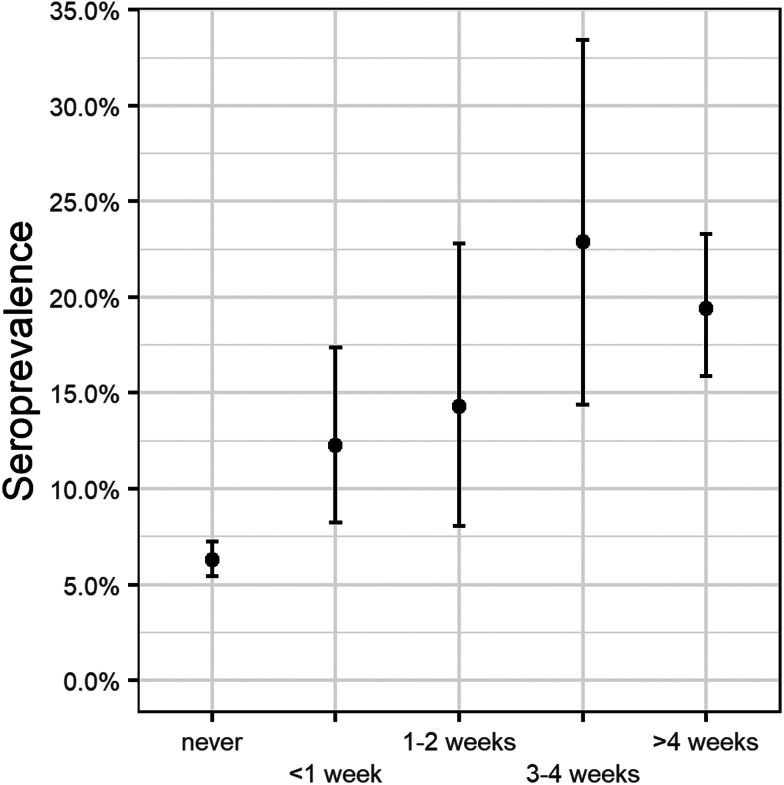
Seroprevalence and exposure time in general ward cohort. Notes: • Only investigating ‘profession_general’ (medical, paramedical and nurse professional categories) • Not taking into account how long the person was working in another ward

Seroprevalence was higher for HCPs working for 3–4 weeks on confirmed COVID-19 wards (22.9%; 95% CI 14.4–33.4) and for those working for more than 4 weeks (19.4%; 95% CI 15.9–23.3) than for staff spending less than a week (12.3%; 95% CI 8.3–17.4) and 1–2 weeks (14.3%; 95% CI 8.0–22.8) on these wards (Fig. 3).

When comparing seroprevalence at the different hospital sites, there was no significant difference for the three larger acute care hospitals (ZNA Middelheim, ZNA Stuivenberg and ZNA Jan Palfijn). Nevertheless, seroprevalences were significantly higher in two chronic care facilities (rehabilitation and geriatric inpatients with dementia) than in the acute care hospitals, respectively 13.0% (95% CI 9.0–18.0) and 22.7% (95% CI 17.9–28.0). In the children's hospital, seroprevalence was significantly lower than in the adult care facilities: 1.7% (95% CI 0.2–6.0).

**Fig. 4.**
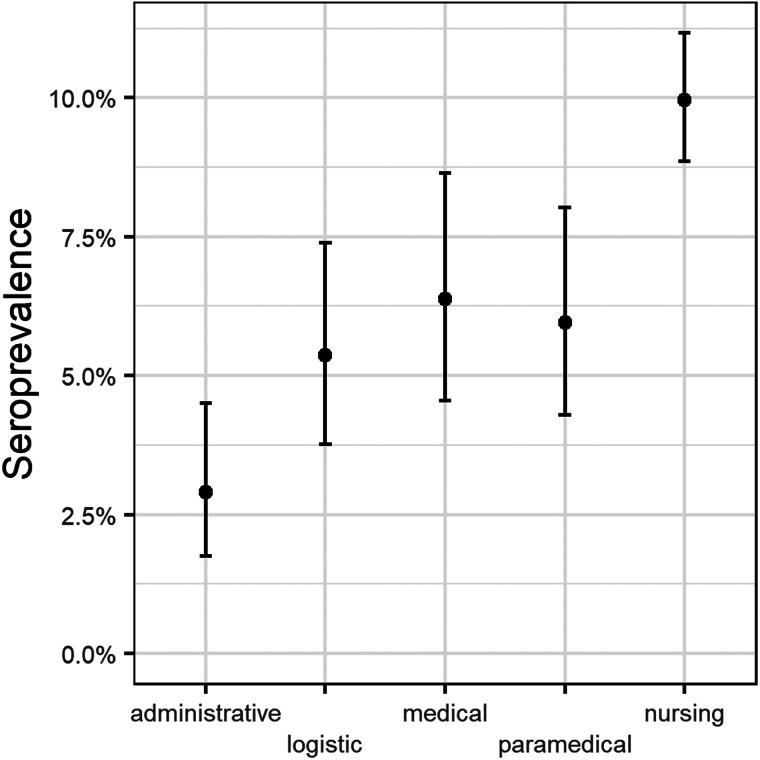
Seroprevalence according to professional categories.

With regard to profession, most participants (*n* = 2660) were nursing staff (50.8%), whereas 596 were medical staff (11.4%), 654 administrative staff (12.5%), 671 (12.8%) paramedical staff and 652 cleaning and technical services staff (12.5%). Nursing staff had a significantly higher seroprevalence (10.0%; 95% CI 8.9–11.2) than all other professional groups, and administrative staff had the lowest seroprevalence (2.9%; 95% CI 1.8–4.5). SARS-CoV-2 serology in paramedical personnel was 6.0% (95% CI 4.3–8.0). Whereas seroprevalence in medical staff was 6.4% (95% CI 4.6–8.7). Physicians in specialty training had a seroprevalence of 11.1% (95% CI 6.7–17.0) whereas attending doctors were positive in 4.7% of cases (95% CI 2.9–7.3) (Fig. 4).

**Fig. 5.**
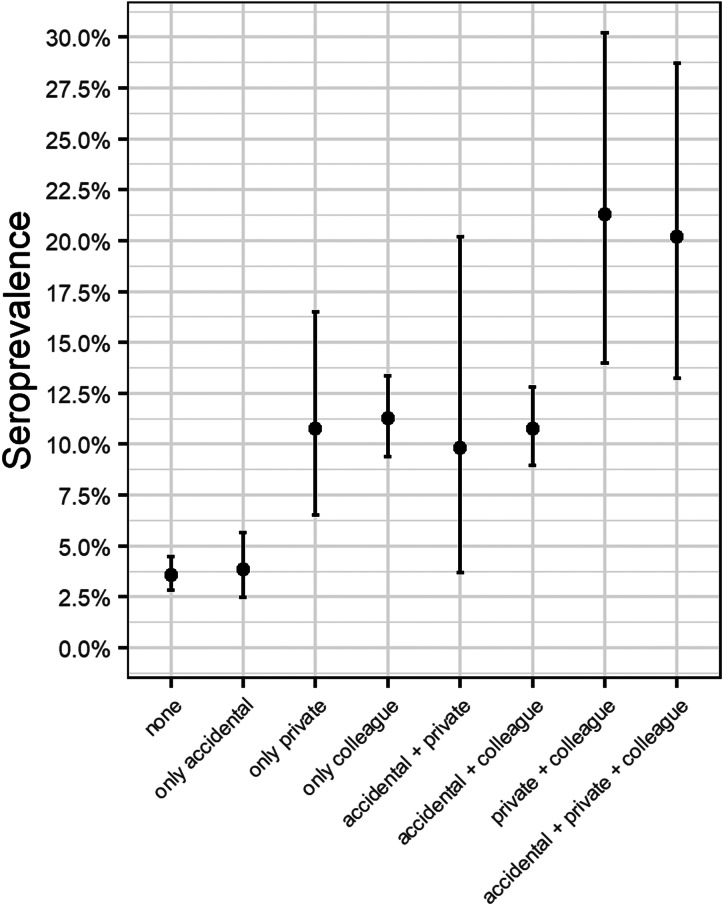
Seroprevalence according to risk factors.

Staff who indicated that a close colleague at work had confirmed COVID-19 (*n* = 2293; 43.8%), had a significantly higher seroprevalence (12.0%; 95% CI 10.7–13.4) than staff not reporting SARS-CoV-2 infected colleagues (4.2%; 95% CI 3.5–5.0) (Fig. 5). The same findings were present in staff that indicated contact with persons in the private or household setting with confirmed or suspected COVID-19 (15.6%; 95% CI 12.3–19.2 *vs*. 6.8%; CI 6.1–7.6) (Fig. 5).

HCPs who reported contacts with positive COVID-19 cases (suspected or confirmed) with adequate protection following hospital guidelines (*n* = 3576; 68.3%) had significantly higher seroprevalences than staff not reporting contacts (*n* = 1657; 31.7%): seroprevalence of respectively 9.4% (95% CI 8.5–10.4) *vs.* 3.7% (95% CI 2.8–4.7).

Staff who reported accidental, unprotected contact (*n* = 1858; 35.5%) with COVID-19 cases (suspected or confirmed) did not have significantly higher seroprevalences than staff who did not have these contacts (*n* = 3375; 64.5%), respectively, 9.0% (95% CI 7.7–10.4) *vs.* 6.8% (95% CI 6.0–7.7).

The final multivariate logistic mixed model included age, profession, working on a COVID-19 ward, indicating a close colleague at work had confirmed COVID-19, contact with confirmed/suspected COVID-19 cases in private setting, at work with adequate protection and at work accidental with an interaction term for the latter two (Table 1). The associations confirm the statements about the comparison of seroprevalences. Medical, paramedical and cleaning and technical staff had lower chances to be seropositive in comparison with nursing staff. An increased chance was observed when indicating a close colleague at work had confirmed COVID-19 and having had contact with confirmed/suspected COVID-19 cases in private setting. In addition, higher age was associated with a lower chance to be seropositive. Including hospital sites as a fixed effect instead of as a random effect did not lead to different results.

**Table 1.** Impact of parameters on seropositivity rate

Parameter	Categories	*N*	Positive (%)	OR (95% CI)^a^	OR (95% CI)^b^
Sex	Female	4174	323 (7.7)	1.00	
	Male	1059	74 (7.0)	0.86 (0.66–1.12)	
Age		5233	397 (7.6)	0.98 (0.97–0.99)	0.99 (0.98–0.99)
Profession	Nursing staff	2660	265 (10.0)	1.00	1.00
	Medical staff	596	38 (6.4)	0.64 (0.44–0.91)	0.65 (0.43–0.94)
	Paramedical staff	671	40 (6.0)	0.60 (0.42–0.84)	0.74 (0.51–0.94)
	Administrative staff	654	19 (2.9)	0.23 (0.14–0.36)	0.40 (0.24–0.66)
	Cleaning and technical services staff	652	35 (5.4)	0.46 (0.31–0.65)	0.63 (0.43–0.93)
Working on a COVID-19 ward	No	3289	172 (5.2)	1.00	1.00
	Yes	1944	225 (11.6)	2.47 (1.98–3.08)	1.73 (1.36–2.20)
Close colleague at work had confirmed COVID-19	No	2940	123 (4.2)	1.00	1.00
	Yes	2293	274 (12.0)	2.63 (2.10–3.31)	1.84 (1.43–2.36)
Contact with confirmed/suspected COVID-19 cases in private setting	No	4783	327 (6.8)	1.00	1.00
	Yes	450	70 (15.6)	2.62 (1.96–3.47)	2.56 (1.91–3.42)
Contact with confirmed/suspected COVID-19 cases at work with adequate protection	No	3375	230 (6.8)	1.00	
	Yes	1858	167 (9.0)	1.22 (0.99–1.51)	
Accidental unprotected contact with confirmed/suspected COVID-19 cases at work accidental	No	1657	61 (3.7)	1.00	
	Yes	3576	336 (9.4)	2.58 (1.95–3.45)	
Contact with confirmed/suspected COVID-19 cases at work: protected and/or accidental and unprotected	No contact	1425	42 (2.9)		1.00
	Only protected contact	1950	188 (9.6)		1.74 (1.19–2.54)
	Only accidental and unprotected contact	232	19 (8.2)		1.44 (0.80–2.59)
	Protected and accidental and unprotected contact	1626	148 (9.1)		1.17 (0.78–1.75)

*N,* number of observations; OR, odds ratio; CI, confidence interval.

a Univariate model using hospital sites as random effect.

b Multivariate model using hospital sites as random effect and with interaction term for contact at work ‘protected’ and ‘accidental and unprotected’.

## Discussion

Among HCPs working in the Antwerp hospital network, SARS-CoV-2 seroprevalence turned out to be 7.6% (95% CI 6.9–8.3). As compared to the general Belgian population for the same testing period, seroprevalence in ZNA personnel was not significantly higher. In the general population aged 20–65 years, the seroprevalence totalled 6.3% (95% CI 5.1–7.7) [19]. Although there was a difference in sex distribution between the study population (female 79.8%, male 20.2%) and the general population (female 50.7%, male 49.3%), statistically significant differences in seroprevalence regarding sex were not observed, neither in our study nor in the survey of the general population [19, 20]. Also, different diagnostic tests were used in both surveys (Roche's Elecsys Anti-SARS-CoV-2 assay in our study *vs*. Euro-Immun's Anti-SARS-CoV-2 IgG assay in the general Belgian population). However, these tests show a fairly equal diagnostic performance, as external validation did not reveal statistically different sensitivities/specificities [21].

Including only medical, nursing and paramedical staff with patient contacts, the seroprevalence was 8.7%. This prevalence was in line with an HCP cohort followed by the Belgian Public Health Institute: for the same testing period, for 850 Belgian HCPs with patient contact from different hospitals throughout the country, the seroprevalence totalled 9.4% (95% CI 6.5–13.4) [22]. Given the results from the country as a whole, our facilities appeared comparable with regard to the use of protective equipment.

Literature on seroprevalence in HCPs is limited, and findings vary substantially: from no additional risk in several serosurveys [5–8] to significantly higher risks in other publications [9–16].

A study in the UK and the United States estimated that frontline healthcare providers had a 3.4-fold higher risk of reporting a SARS-CoV-2-positive test (PCR or antibody test), adjusted for the likelihood of having a test, than people living in the general community [9].

The European Center for Disease Prevention and Control (ECDC) analysed case-based surveillance data from a total of 124.796 reported cases with known healthcare worker status from 15 countries in the EU/EAA and the UK. The overall percentage of HCPs among COVID-19 cases was 23% (*n* = 43 774/188 693), with a country median of 14% (range 1–59%) [16].

In our opinion, one must be very careful in coming to conclusions regarding the low risk of exposure in HCPs reflected in some of the publications. Firstly, the timing of serology-testing is crucial. Indeed, we carried out our testing in the first weeks following the first pandemic wave when Belgium was in full lockdown and social contacts were very limited outside the hospital (Fig. 1). Testing too early in an epidemic wave leads to potentially low cumulative exposure of hospital staff to positive individuals, which was already suggested by Mansour *et al*. [13]. Waning immunity and an increase in social contacts because of relaxation of measures [23] have a negative impact on correct interpretation of seroprevalence numbers in HCPs, due to delay in testing. Secondly, defining groups in the frontline accurately is crucial: when the cumulative time spent on the COVID-19 wards is limited, additional risks may become undetectable. Thirdly, the type of test performed is crucial. Highly sensitive and highly specific tests are needed for correct interpretation. If one or more of those items is lacking, results may be falsely reassuring, as might have been the case in the article by Steensels *et al*. and Jacob *et al*. [5, 6].

Seroprevalence in ZNA HCPs working in confirmed COVID-19 wards was significantly higher than in other HCPs (Fig. 2). For the confirmed COVID-19 wards, we found a time-related trend, although statistically insignificant, towards higher seroprevalences (Fig. 3). One possible explanation for these findings may be patient behaviour. Patients do not always wear masks (in line with hospital guidelines, e.g. geriatric patients suffering from dementia or delirium). The importance of behavioural and clinical characteristics is also reflected in the significantly higher seroprevalences in two chronic care hospitals with a majority of elderly people.

We could not identify factors, except for above-mentioned study-design confounders, explaining why this higher exposure in general COVID-19 wards was not withheld by some other study groups [5, 6]. The level of adherence to the guidelines and/or infrastructural factors (for example ventilation) might have been underlying factors. On the other hand, our findings were consistent at all hospital sites.

In our opinion, even with predetermined PPE, it is difficult to attain zero risk exposure to the SARS-CoV-2 virus in COVID-19 units. This is also reflected by the high association of seroprevalence in hospital staff who have been in contact with COVID-19 cases and have been using PPE, in line with hospital guidelines. Surprisingly, this association was not found in the large (>20.000 HCP) multi-centre study in three states of the US [5] (Fig. 5).

Working on COVID-19 ED and ICU wards was not related to significantly higher seroprevalences. For the ED, although the numbers of patient contacts may be higher, cumulative time of exposure of HCPs to COVID-19 positive patients is far lower than exposure on a hospital ward. The contacts may also be less intense (e.g. hygienic care of patient). Another hypothesis for the lower seroprevalence in HCPs working in COVID-19 ICUs could be the lower viral loads in these patients than in patients on general COVID-19 wards (unpublished ZNA data), the higher use of closed-loop circuit ventilation (with appropriate heat and moisture exchange filters) and the higher nurse-to-patient ratio, resulting in lower ‘at risk exposure time’ [24].

In our study, there was a clear association of professional category with antibody positivity (nurses having highest rates), again consistent with some studies or epidemiological reports, but conflicting with other surveys [5, 15, 25] (Fig. 4).

As regard to age, higher seroprevalences were found in the younger adult age groups. This was also found in other surveys: Jacob *et al*. showed community-associated increased risks of seropositivity in HCP being younger than 30 years [5]. Also in the general population of Antwerp, the age group of 21–30 years old had significantly higher rates of viral carriership as compared to the age group of 51–60 years old [26]. Beside the community-associated factor, it might also be that elder HCPs were more experienced and adherent with hospital procedures.

Interestingly, positive cases in HCPs in our hospital were clustered. This finding was most overt in the nurse group. Although our hospital was quick to recognise the role of asymptomatic individuals, HCPs may have been insufficiently aware of that invisible risk. Also, testing policy in the first viral wave mainly focused on symptomatic HCPs.

As a result of this survey, our communication strategy was to advise all HCPs to be more sensitive to the risks of SARS-CoV-2 transmission between colleagues. Also, a stringent testing and contact-tracing policy has been put in place with special attention to the health care workers employed in general COVID-19 departments.

Our study shows several limitations. This subjective questionnaire (in which there was an indication that an HCP had a close colleague with confirmed COVID-19) was not externally validated by the number of objectively determined COVID-19 cases among the total hospital staff. No correction was made for an individual's total working time on one COVID-19 ward, in relation to their working time on another COVID-19 ward. Both factors could have impacted our results.

In conclusion, our analyses show that working on confirmed COVID-19 wards led to a higher risk of being exposed to SARS-CoV-2 than not working on COVID-19 wards. Risks for staff working in ED departments and ICU were within the estimated ranges of the general Belgian population. The risk was clearly related to professional categories with nurses belonging to the highest risk group. More positive SARS-CoV-2 serology cases were documented when contacts with infected colleagues or household members were reported.
